# Jhaukhel-Duwakot Health Demographic Surveillance Site, Nepal: 2012 follow-up survey and use of skilled birth attendants

**DOI:** 10.3402/gha.v8.29396

**Published:** 2015-12-21

**Authors:** Bishnu P. Choulagai, Umesh Raj Aryal, Binjwala Shrestha, Abhinav Vaidya, Sharad Onta, Max Petzold, Alexandra Krettek

**Affiliations:** 1 Department of Community Medicine and Public Health, Institute of Medicine, Tribhuvan University, Kathmandu, Nepal; 2 Department of Internal Medicine and Clinical Nutrition, Institute of Medicine, Sahlgrenska Academy, University of Gothenburg, Gothenburg, Sweden; 3 Department of Community Medicine, Kathmandu Medical College, Kathmandu, Nepal; 4 Health Metrics, Sahlgrenska Academy, University of Gothenburg, Gothenburg, Sweden; 5 School of Public Health, Faculty of Health Sciences, University of the Witwatersrand, Johannesburg, South Africa; 6 Department of Biomedicine and Public Health, School of Health and Education, University of Skövde, Skövde, Sweden; 7 Department of Community Medicine, Faculty of Health Sciences, UiT The Arctic University of Norway, Tromsø, Norway

**Keywords:** health demographic surveillance site, JD-HDSS, health systems research, skilled birth attendants, public health, Nepal

## Abstract

**Background:**

Estimates of disease burden in Nepal are based on cross-sectional studies that provide inadequate epidemiological information to support public health decisions. This study compares the health and demographic indicators at the end of 2012 in the Jhaukhel-Duwakot Health Demographic Surveillance Site (JD-HDSS) with the baseline conducted at the end of 2010. We also report on the use of skilled birth attendants (SBAs) and associated factors in the JD-HDSS at the follow-up point.

**Design:**

We used a structured questionnaire to survey 3,505 households in the JD-HDSS, Bhaktapur, Nepal. To investigate the use of SBAs, we interviewed 434 women who had delivered a baby within the prior 2 years. We compared demographic and health indicators at baseline and follow-up and assessed the association of SBA services with background variables.

**Results:**

Due to rising in-migration, the total population and number of households in the JD-HDSS increased (13,669 and 2,712 in 2010 vs. 16,918 and 3,505 in 2012). Self-reported morbidity decreased (11.1% vs. 7.1%, respectively), whereas accidents and injuries increased (2.9% vs. 6.5% of overall morbidity, respectively). At follow-up, the proportion of institutional delivery (93.1%) exceeded the national average (36%). Women who accessed antenatal care and used transport (e.g. bus, taxi, motorcycle) to reach a health facility were more likely to access institutional delivery.

**Conclusions:**

High in-migration increased the total population and number of households in the JD-HDSS, a peri-urban area where most health indicators exceed the national average. Major morbidity conditions (respiratory diseases, fever, gastrointestinal problems, and bone and joint problems) remain unchanged. Further investigation of reasons for increased proportion of accidents and injuries are recommended for their timely prevention. More than 90% of our respondents received adequate antenatal care and used institutional delivery, but only 13.2% accessed adequate postnatal care. Availability of transport and use of antenatal care was associated positively with institutional delivery.

Countries in Asia and Africa frequently lack reliable national data. Therefore, many national and global estimates of population health have relied on extrapolation and modeling approaches using limited data (1). Byass and colleagues reported that detailed local data, such as those produced by health demographic surveillance sites (HDSS), are likely to adequately reflect national data, making them suitable for generalizing into policy (2). This finding is very important in countries like Nepal, where the scarcity of reliable and accurate longitudinal data inhibits development of evidence-based policy (2).

Estimates of disease burden in Nepal are based on cross-sectional studies, including a national census every 10 years, a national demographic and health survey every 5 years, and additional studies conducted by the Ministry of Health and Population and other agencies. These data provide inadequate epidemiological information to support critical decisions by health planners, policy makers, and managers (3).

Although Nepal's vital records system gathers continuous data on births, deaths, and marriage formation and dissolution, coverage is poor, registering only just over one-third of all births (4). Compared to periodic retrospective surveys, an HDSS ensures more accurate records over time, especially in a population where education levels are low (4).

In 1996, a household registration system including demographic surveillance system methodology and focusing on migration issues was established in Chitwan, a southern district in central Nepal (4). We established the Jhaukhel-Duwakot HDSS (JD-HDSS) in 2010 in the Jhaukhel and Duwakot Village Development Committees (VDCs) of Bhaktapur District (3).

In countries with a weak vital registration system, an HDSS can provide data on vital events and a sampling frame for health research (5). We conducted a study on smoking susceptibility among adolescents in the JD-HDSS in October–November 2011 (6) and another study on cardiovascular health knowledge, attitude, and behavior in September–November 2011 (7). We also explored community experiences and perceptions about the causes and prevention of cardiovascular disease among people with cardiometabolic conditions (8).

As Nepal strives to attain the Millennium Development Goals (MDGs), the government is committed to increasing the proportion of births attended by skilled birth attendants (SBAs) (9). Although SBA coverage has increased nationally, from 9% in 1996 to 36% in 2011 (10, 11), Nepal still lags behind the 60% compliance target set by the World Health Organization for 2015 (9). Women's use of SBAs is unevenly distributed between rural and urban areas. Indeed, the recent Nepal Demographic and Health Survey showed that 27.9% of deliveries in urban areas and 66.7% of deliveries in rural areas occur at home (12). With less than 6 months remaining to achieve the MDG targets, the issues surrounding SBA services remain pertinent in the post-MDG health and development agenda.

An HDSS starts with a baseline survey and then conducts a follow-up survey to gather health and demographic indicators (i.e. fertility, migration, morbidity, and mortality) (13). Baseline surveys are useful for examining the access, quality, and utilization of health-care services (14). This study compares the health and demographic indicators at the end of 2012 in the JD-HDSS with the baseline survey conducted at the end of 2010.

Earlier studies on SBA services focused mostly on rural areas (15–19). Our follow-up survey assessed the use of SBA services and associated factors in the peri-urban JD-HDSS.

## Methods

### Study site and population

The Duwakot and Jhaukhel VDCs lie in the mid-hills of Bhaktapur District adjacent to Kathmandu, the capital city of Nepal. Both VDCs represent prototypical urbanizing villages near Nepal's larger towns. We previously described the setting of the JD-HDSS (3). We conducted a complete enumeration of the population residing in the surveillance site. Our follow-up survey on SBA usage included all married women of reproductive age who had delivered a baby during the two years prior to data collection.

### Recruitment and training of field staff

A core local management committee comprising four PhD students and a coordinator planned, organized, and oversaw HDSS activities. We recruited and trained 18 enumerators and 4 supervisors to execute the field survey. Enumerator training included instructions for conducting data collection and an explanation of each section of the collection tools that were developed from the baseline questionnaire. All enumerators and supervisors received a field manual that provided specific instructions on how to complete the questionnaire interview forms. Enumerator training also included a pretest of the collection tools.

### Data collection and field supervision

We based the follow-up questionnaire on our original baseline survey (3). In addition to socioeconomic information, demographic parameters, morbidity, health-seeking behaviors, and environmental factors, we used a separate structured questionnaire to determine use of SBAs and associated factors. Enumerators recorded any respondent illnesses that occurred in the 4 weeks preceding the survey. We coded each response for data entry.

Nine groups of enumerators (two enumerators per group) collected household data. Four field supervisors (two per VDC) were available in the field during the entire collection period to supervise and support the enumerators. The field supervisors regularly reported the status of data collection to the PhD students.

The PhD students (BC, BS) supervised data collection during regular field visits. Several meetings with the enumerators and field supervisors helped identify problems and correct errors. The PhD students’ academic supervisors (AK, MP, SO) provided overall guidance.

### Data management and analysis

Public health graduates entered the data using EpiData software, version 3.1. We checked the data entry process regularly and discussed with data entry operators any problems they faced during data entry. Then, data were transferred into IBM SPSS Statistics, version 20, for analysis.

The fertility and mortality indicators were calculated based on the measurement of occurrence of such events within 1 year preceding the survey. Those residents who moved into the surveillance site at least 3 months prior to data collection were considered in-migrants, whereas those who left the surveillance site for 3 months or longer were considered out-migrants.

Data analysis involved both descriptive (percentage, mean, standard deviation) and inferential statistics (95% CI for differences, logistic regression). We compared demographic parameters, morbidity, health-seeking behaviors, and environmental factors with data from the baseline survey.

Principal component analysis (20) determined household economic status by calculating a wealth index based on household assets. The wealth index was computed using the first principal component and based on the availability of 17 kinds of household assets.

We employed multivariate logistic regression analysis to assess the association of antenatal, delivery, and postnatal care with independent variables (education, occupation, ethnicity, age, wealth quintile, and means of transport). To check collinearity, we calculated the variance inflation factor (VIF) and detected no problem among the independent variables (highest VIF, 1.15) that would prevent their inclusion in analysis. Multivariate logistic regression analyses included all independent variables that were significant at the 15% (21) level in the bivariate logistic regression analyses.

### Ethical considerations

After explaining the nature of the study, its rationale, and the extent of participant involvement, the enumerators sought verbal informed consent from every participant. The Nepal Health Research Council granted ethical approval for this study. We also briefed local administrative authorities, health personnel, and political leaders about the objectives of our study and obtained their verbal permission to conduct the survey. To ensure confidentiality, all data were secured in the HDSS office at Jhaukhel, Bhaktapur.

## Results

### Sociodemographic indicators

Data from Nepal's 2011 national census indicates that the JD-HDSS covers 5.55% of the total population of Bhaktapur District (22). Between the baseline and follow-up surveys (2010 and 2012, respectively), the total population of the JD-HDSS increased by 23.7% (Table 1). Mean age in the follow-up survey was similar for both sexes (29.5±18.6 years for males and 29.9±18.7 years for females). Compared to the baseline survey, the proportion of children <5 years of age increased from 5.8 to 6.3%, as did the proportion of people aged ≥70 years (3.0 to 3.3%).

**Table 1:** Household size, population size, and sex ratio in the Jhaukhel-Duwakot Health Demographic Surveillance Site during 2010 and 2012

Variable	2010	2012
Total households	2,712	3,505
Total population	13,669	16,918
Males	6,868	8,516
Females	6,801	8,402
Sex ratio (males per female)	1.010	1.002
Sex ratio at birth (males per female)	NA	1.64:1
Median family size	5.0 (1–21)	4.0 (1–18)

Note: Data are shown as a comparison of general characteristics of the study population between the baseline survey in 2010 (3) and follow-up survey in 2012. NA, not available.

At follow-up, one-third (33.1%) of the population belonged to the Newar caste, followed by Chhetri (31.6%) and Brahmin (21.8%). Disadvantaged Janajati (Tamang, Magar, and Rai) and lower caste (Dalit) accounted for 8.7 and 2.8% of the total population, respectively. Similar to the finding at baseline (97%) the predominant religion was Hindu (95.6%), followed by Buddhism (2.6%) and Christianity (1.7%). The proportion of illiterate people aged ≥6 years decreased (18.2% at baseline vs. 16.4% at follow-up, data not shown). The proportion of illiterate people aged ≥15 years was 19.4%.

More than one-third of the population (35.4%) had completed secondary-level education (Grade 10), and 1.8% had completed master's level education. Although the percentage of people working in agriculture remained unchanged (10.6%), the population working in the service sector decreased (20.0% at baseline vs. 15.5% at follow-up). The proportion of unemployed people decreased (2% at baseline vs. 1% at follow-up).

### Fertility

Although we determined a small increase in the crude birth rate (9.7 vs. 11.7 per 1,000 population), the change was not statistically significant (Table 2). The mean age of girls at marriage increased (18.4 years at baseline vs. 19 years at follow-up); the mean age at first childbirth was 20 years in both surveys.

**Table 2:** Vital statistics from the Jhaukhel-Duwakot Health Demographic Surveillance Site, 2010 and 2012

Variable	2010	2012	Difference	Lower CI	Upper CI
Fertility					
Crude birth rate (per 1,000 population)	9.7	11.7	2.0	−2.19	6.39
General fertility rate (per 1,000 female population, 15–49 yrs.)	32.0	38.0	6.0	−27.47	39.47
Institutional delivery (%)	88.7	93.1	4.4	1.11	7.69*
Mortality					
Crude death rate (per 1,000 population)	3.9	3.8	0.1	−2.01	1.81
Infant mortality rate^a^	–	–			
Maternal mortality ratio^a^	–	–			
Morbidity					
Illness (%)	11.1	7.1	−0.4	0.71	7.29*
Migration					
In-migration (%)	2.3	10.5	−8.2	−11.49	−4.91*
Out-migration (%)	1.4	2.7	−1.3	−4.59	1.99

Note: Data show a comparison of vital statistics between the baseline (3) and follow-up survey. CI, confidence interval;

* *p*<0.05;

a no infant or maternal deaths were reported during the survey period.

### Migration

With 1,783 in-migrants (10.5%) in the follow-up survey, in-migration to the JD-HDSS increased more than fourfold (2.3% at baseline vs. 10.5% at follow-up), which was statistically significant (Table 2). The main reasons for in-migration were service (33.3%), business (11.7%), and study (8.9%). The proportion of males was higher than females among the in-migrants (63.9% vs. 36.1%). Out-migrants at follow-up totaled 462 (2.7%), which was greater but not significant compared to baseline (1.4%) (Table 2). The main reasons for out-migration were service (57.0%) and study (21.9%). More males than females out-migrated from the surveillance site (78.8% vs. 21.2%).

### Mortality, morbidity, and health behaviors

The crude death rate was almost the same (3.9 and 3.8 per 1,000 population, respectively) in both surveys. In the follow-up, a total of 65 deaths were reported with 57% male and 43% female deaths. Both surveys recorded no infant and maternal deaths.

Reported morbidity decreased significantly (Table 2). Although the position of top two morbidities (respiratory diseases and fever) remained unchanged, respiratory diseases increased and fever decreased in proportion to the overall morbidity (Table 3). The proportion of accidents and injuries contributing to overall morbidity increased (2.9% vs. 6.5%, respectively). Gastrointestinal problems increased (13.9% vs. 18.1%), propelling them to the third leading cause of morbidity in the follow-up survey. Additionally, morbidity resulting from the four main non-communicable diseases (heart disease, hypertension, cancer, and diabetes) declined (12% vs. 5.7%, respectively).

**Table 3:** Top morbidity conditions reported in the Jhaukhel-Duwakot Health Demographic Surveillance Site, 2010 and 2012

	Type of morbidity (%)	
	
Ranking	2010 (*N*=1,517)	2012 (*N*=1,142)
1	Respiratory diseases (41.9)	Respiratory diseases (48.4)
2	Fever (41.1)	Fever (24.5)
3	Headache and dizziness (16.7)	Gastrointestinal problems (18.1)
4	Bone and joint pain (14.4)	Bone and joint pain (13.4)
5	Gastrointestinal problems (13.9)	Accidents and injuries (6.5)
6	Heart diseases, including hypertension (8.8)	Heart diseases, including hypertension (4.9)
7	Accidents and injuries (2.9)	Skin problems (2.9)
8	Skin problems (2.9)	Headache and dizziness (2.9)
9	Diabetes mellitus (2.6)	Uterine and vaginal problems (2.0)
10	Dental problems (1.6)	Eye problems (1.6)

Note: Results are shown for self-reported illnesses that occurred within 4 weeks prior to the baseline (3) and follow-up surveys. Multiple responses were recorded if more than one cause was provided.

Regarding treatment-seeking behavior, more than one in five respondents (22.7%) in the follow-up survey had visited a private clinic and 16.9% had used the district hospital of Bhaktapur. Another 14.2% visited teaching hospitals run by Nepal Medical College and Kathmandu Medical College in Kathmandu, and 5.4% visited local pharmacy shops for treatment.

The follow-up survey also assessed smoking behavior and alcohol consumption among people aged ≥18 years. Although overall smoking prevalence was similar (15% at baseline vs. 15.5% at follow-up, data not shown), smoking in males increased (20% at baseline vs. 23% at follow-up). Additionally, the follow-up survey showed that 12% of the people currently consume alcohol (15.5% male vs. 8.5% female).

### Sociodemographic characteristics of women participating in the SBA study

Our study of SBA services included 434 women (median age 26 years) in the JD-HDSS who had delivered a baby within 2 years prior to the survey. Most (90.1%) were 20–34 years of age, and 5.8% were <20 years (Table 4). The predominant ethnicity was Brahmin/Chhetri (45.5%), followed by Newar (38.8%).

**Table 4:** General characteristics of women participating in the study of utilization of skilled birth attendants at the Jhaukhel-Duwakot Health Demographic Surveillance Site (*N*=434)

Variables	*N*	%
Age groups (years)		
<20	25	5.8
20–24	140	32.3
25–29	198	45.6
30–34	53	12.2
≥35	18	4.1
Ethnicity		
Newar	166	38.8
Brahmin/Chhetri	196	45.5
Disadvantaged Janajati and Dalit	66	15.4
Education		
Illiterate^a^	94	21.7
Informal^b^	51	11.8
Primary^c^	49	11.3
Secondary^d^	135	31.1
Intermediate and above^e^	105	24.1
Occupation		
Agriculture	65	15.0
Service	31	7.2
Business	28	6.5
Wage laborer	17	3.9
Housewife	292	67.4
Wealth quintile		
Lowest	88	20.3
Second	91	21.0
Middle	81	18.7
Fourth	89	20.4
Highest	85	19.6

Note: Results are shown for 434 women who delivered a baby within 2 years prior to the survey.

a Unable to read and write;

b learning not connected to formal schools;

c Grades 1–5;

d Grades 6–10;

e Grades 11 and above.

Although more than one in five women (21.7%) were illiterate, nearly one in four women (24.1%) had achieved intermediate-level education or above. About two-thirds (67.4%) were housewives, 15% worked in agriculture, and 7.2% worked in the service sector (Table 5).

**Table 5:** Maternal health characteristics of women participating in the study of utilization of skilled birth attendant services at the Jhaukhel-Duwakot Health Demographic Surveillance Site

Variables	*N*	%
Antenatal care visits (frequency)		
Inadequate	40	9.2
Adequate (four or more visits)	394	90.8
Utilization of delivery services		
No	26	6.9
Yes	353	93.1
Postnatal care visits (frequency)		
Inadequate	334	86.8
Adequate (three or more visits)	51	13.2
Distance to health facility		
>30 min	315	73.8
≤30 min	112	26.2

Note: Results are shown for 434 women who delivered a baby within 2 years prior to the survey.

### Utilization of SBAs

Almost all women (97.2%) had attended at least one antenatal care visit and 90.8% completed the adequate four or more visits (23). Utilization of institutional delivery service was 93.1%, and 13.2% of the women completed three postnatal care visits. Nearly three-fourths of respondents (73.8%) reported that their walking time to the nearest health facility was more than 30 min.

We used multivariate logistic regression analysis to assess the association of antenatal, delivery, and postnatal care services with background variables (Tables 6–8). Ethnicity was associated with adequate antenatal care visits, whereas other independent variables (i.e. education, wealth, and means of transport) were not significantly associated with such use. Newar and Brahmin/Chhetri women were 5.0 (95% CI: 2.06–12.28) and 5.7 times (95% CI: 2.22–14.83) more likely to access adequate antenatal care services compared to disadvantaged Janajati and Dalit women.

**Table 6:** Determinants of antenatal care service utilization at the Jhaukhel-Duwakot Health Demographic Surveillance Site, Bhaktapur, Nepal

	Confidence interval			
	
Characteristics	Odds ratio	Lower	Upper	*p*
Education				
Illiterate	1			
Informal education	0.34	0.11	1.07	0.065
Primary school	0.79	0.22	2.87	0.714
Secondary school	1.65	0.50	5.51	0.415
Intermediate and above	0.60	0.20	1.76	0.350
Wealth quintile				
Lowest	1			
Second	0.85	0.33	2.16	0.725
Middle	0.85	0.38	2.55	0.772
Fourth	2.68	0.78	9.17	0.117
Highest	2.34	0.58	9.38	0.231
Ethnicity				
Disadvantaged Janajati and Dalit	1			
Newar	5.03	2.06	12.28	<0.001
Brahmin/Chhetri	5.73	2.22	14.83	<0.001
Transport				
Walk to health facility	1			
Use means of transport	1.1	0.51	2.37	0.816

Antenatal care utilization (less than four times and four or more times). Note: The association between antenatal care utilization and various independent variables is shown.

**Table 7:** Determinants of delivery service utilization at the Jhaukhel-Duwakot Health Demographic Surveillance Site, Bhaktapur, Nepal

	Delivery service utilization			
	
		Confidence interval		
		
Characteristics	Odds ratio	Lower	Upper	*p*
Education				
Illiterate^a^	1			
Informal education^b^	0.41	0.60	2.85	0.369
Primary school^c^	0.47	0.07	3.39	0.456
Secondary school^d^	0.39	0.08	1.97	0.252
Intermediate and above^e^	1.69	4.41	62.35	0.586
Distance				
≥30 min	1			
≤30 min	0.81	0.26	2.55	0.723
Antenatal care visit				
Inadequate	1			
Adequate	21.17	5.53	81.00	<0.001
Ethnicity				
Disadvantaged Janajati and Dalit	1			
Newar	4.01	0.80	20.02	0.091
Brahmin/Chhetri	0.45	0.11	1.79	0.257
Means of transport				
Walk to health facility	1			
Use means of transport	16.60	4.41	62.35	<0.001

Note: Results are shown for the association between delivery care utilization and various independent variables.

a Unable to read and write;

b learning not connected to formal schools;

c Grades 1–5;

d Grades 6–10;

e Grades 11 and above.

**Table 8:** Determinants of postnatal care service utilization at the Jhaukhel-Duwakot Health Demographic Surveillance Site

	Utilization of postnatal care			
	
		Confidence interval		
		
Characteristics	Odds ratio	Lower	Upper	*p*
Occupation				
Agriculture	1			
Service	4.82	1.18	19.62	0.028
Business	3.47	0.75	16.12	0.112
Wage laborer	2.88	0.44	18.86	0.270
Housewife	2.55	0.85	7.62	0.094
Wealth quintile				
Lowest	1			
Second	3.21	0.82	12.52	0.093
Middle	3.24	0.75	13.94	0.115
Fourth	7.03	1.87	26.45	0.004
Highest	7.12	1.84	27.95	0.005
Ethnicity				
Disadvantaged Janajati and Dalit	1			
Newar	0.44	0.18	1.08	0.073
Brahmin/Chhetri	0.34	0.14	0.84	0.019

Note: Results are shown for the association between postnatal care utilization and various independent variables.

Adequacy of antenatal care visits (four or more visits) and use of transport were positively associated with institutional delivery. Women who received adequate antenatal care were 21 times more likely to use institutional delivery services. Women with access to transport (e.g. bus, taxi, or motorcycle) were 16.6 times more likely to use institutional delivery services.

Adequate postnatal care visits (24) were associated with occupation, wealth quintile, and ethnicity. Women who worked in the service sector were five times more likely to attend an adequate number of postnatal care visits compared to those who worked in agriculture. Women in the fourth and fifth wealth quintiles were seven times more likely to use postnatal care (24) services compared to women in the poorest quintile. Brahmin/Chhetri women were 0.34 times less likely to attend an adequate number of postnatal care (24) visits compared to disadvantaged Janajati and Dalit women.

## Discussion

Our initial baseline in the JD-HDSS was conducted at the end of 2010 (3); the follow-up survey occurred at the end of 2012. The follow-up survey also introduced an assessment of women's use of SBA services.

### Sociodemographic findings

The total population of the JD-HDSS increased 23.8% (13,669 in 2010 vs. 16,918 in 2012), largely due to increased in-migration. This rapidly growing and urbanizing area is moving toward an urban lifestyle (6), possibly explaining the increased level of in-migration. Nepal's recent census shows that rural-to-urban migration is common, including in Bhaktapur, a predominantly urban district that received 31% in-migrants in 2001–2011 (25). This population growth also explains the increased number of households in the JD-HDSS.

**Fig. 1:**
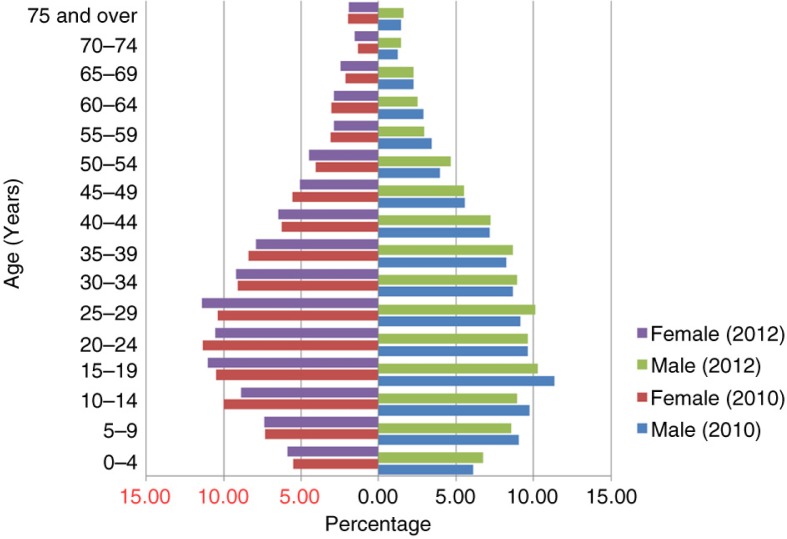
An overlay of the population pyramids in the Jhaukhel-Duwakot Health Demographic Surveillance Site from the baseline and follow-up surveys. The figure shows an overlay of age- and sex-wise population structures that existed at baseline (December 2010) and follow-up (December 2012) in the Jhaukhel-Duwakot Health Demographic Surveillance Site, Bhaktapur, Nepal.

Both surveys show the population structure of the JD-HDSS as a constrictive pyramid (Fig. 1). In Nepal's national population pyramid, the population gradually increases in the age groups 0–4 years, 5–9 years, and 10–14 years; it then begins to decline in the 15–19-year-old age group (25). In contrast, the JD-HDSS population gradually increases up to the 25–29-year-old age group and starts to decline in the 30–34-year-old age group, revealing declining fertility and mortality rates in this peri-urban setting. Although the fertility rate is declining nationally, the crude birth rate is lower in the JD-HDSS (22 vs. 11.7 per 1,000, respectively) (25). The most recent national census (2011) showed lower fertility in urban areas (26). Our peri-urban JD-HDSS shows similar findings.

### Mortality, morbidity, and health behaviors

Compared to the latest national census (2011), the crude death rate in JD-HDSS is about half of the national level (3.8 vs. 7.3 per 1,000 population, respectively) (25). Our surveys recorded no maternal and infant mortalities, possibly due to the high utilization of antenatal care, institutional delivery, and postnatal care services. In HDSS, maternal mortality is rare and, thus, difficult to determine in small sample sizes (27).

Increasing population, rapid urbanization, industrialization, migration, and changing lifestyles in Nepal have resulted in increased violence, injuries, and disabilities. Injuries account for about 8% of all deaths (28). Most accidents and injuries result from road-traffic accidents, interpersonal violence, poisoning, falls, and fires, and the highest proportion of road-traffic accidents occurs in the central region (28). In the JD-HDSS, the proportion of accidents and injuries has increased (2.9% at baseline vs. 6.5% at follow-up), possibly due to rapid urbanization and increasing population and in-migration.

Morbidity in the total population decreased (11.1% at baseline vs. 7.1% at follow-up) (Table 2), as did the proportion of non-communicable diseases (12% at baseline vs. 5.7% at follow-up). Although we conducted the survey during the same season of the year (October–December), increasing urbanization and health service availability and use could explain this decline in overall morbidity.

Studies on non-communicable diseases in various settings in Nepal report varying prevalence. A nationally representative hospital-based study reports that 31% of all admitted cases suffer from non-communicable diseases (29). In Eastern Nepal, the prevalence of coronary heart disease is 5.7% (30), whereas the national prevalence of hypertension in urban adults is 20% (31). Likewise, the prevalence of diabetes and impaired fasting glucose is 14.2 and 9.1%, respectively, in an urban population (32). Others suggest that HDSS sites may have better health indicators compared to other populations because repeated data collection activities could function as a passive intervention (5). In the JD-HDSS, we have conducted other health-related research, including health camps, which might have contributed to reduced disease prevalence and increased awareness of health-related behaviors. The self-reported decrease in morbidity shown during follow-up should be interpreted cautiously because we measured the occurrence of illness by recall method, possibly introducing recall bias.

Tobacco-smoking behavior was unchanged at follow-up, although smoking among males increased (20% at baseline vs. 23% at follow-up). Smoking prevalence was lower in the JD-HDSS compared to other populations in Nepal, such as college students from Western Nepal (34.2%) (33) and males in Dharan Municipality (42.7%) (34). Similarly, alcohol consumption was more prevalent among males (15.5%) than females (8.5%). Nationally, 41% of the population used alcohol during the past year (48.3% male and 27.7% female) (35). Another study from Eastern Nepal reports that 16.6% of women aged ≥15 years consume alcohol (36).

### Use of SBAs

We estimated the current use of antenatal, delivery, and postnatal care in the JD-HDSS and assessed the factors that influence such use. Antenatal care visits are an important platform for educating pregnant women and encouraging them to deliver their babies in a health facility. In managing pregnant women without evidence of pregnancy-related complications, medical conditions, or major health-related risk factors, the WHO recommends a minimum of four antenatal-care visits: 1) before 16 weeks, 2) at 24–28 weeks, 3) at 30–32 weeks, and 4) at 36–38 weeks (23). In the JD-HDSS, 90.8% of women attended at least four antenatal care visits, exceeding the national statistics (71.8% for urban areas) reported in the Nepal Demographic and Health Survey 2011 (12). However, the use of antenatal care differed significantly among ethnic groups. Advantaged ethnic groups (Newar and Brahmin/Chhetri) were 5.0 and 5.7 times more likely, respectively, to use such services. In a systematic review of 28 published research articles from low- and middle-income countries, nine studies showed an association of ethnicity and religion with the use of antenatal care services (37).

Antenatal care offers an important opportunity to teach pregnant women about the danger signs of pregnancy, enables them to recognize early symptoms, and if needed instructs them to go to a health facility as soon as possible (15). In our study, adequate antenatal care visits and access to transport were strongly associated with the use of delivery care services. Our previous study in three districts of mid- and far-western Nepal (15) and a study by Dhakal and colleagues in two VDCs near Kathmandu (18) also demonstrated an association between adequate antenatal care visits and use of delivery services. Similar findings have been reported in studies from Ethiopia, Laos, and Bangladesh (38–40). Contrary to other study findings, mostly from rural areas (19–37), the association of distance with the use of delivery care was not significant in our study, possibly due to increasing availability of transport options in the rapidly urbanizing HDSS. Increased distance from a health facility decreases the use of delivery care, but it is also difficult to determine (41).

Our finding that access to transport strongly predicts the likelihood that women will seek delivery care concurs with our previous quantitative and qualitative studies in mid- and far-Western Nepal (15, 16) and a study in the rural Kavre District of Nepal (42). Studies from Afghanistan and Pakistan report that access to transport associates positively with the use of delivery care (43, 44).

Promotion of postnatal care services contributes importantly to maternal and neonatal health and reduces maternal and neonatal mortality (45). Nepal's Ministry of Health recommends that women receive at least three postnatal checkups, the first within 24 h of delivery, the second on the third day following delivery, and the third on the seventh day following delivery (24). We determined that only about one in seven (13.2%) women completed the recommended three postnatal care visits within 7 days after delivery. Only one-fifth of women in a Western Nepal district accessed postnatal care from healthcare workers (46), suggesting lower utilization of such services compared to antenatal and delivery care. An Ethiopian study reports that only 2.9% of women completed three or more postnatal care visits, suggesting lack of time, long distance to a provider, and lack of guardians for childcare as reasons for low utilization of postnatal care services (47). Other reasons include believing that postnatal visits are not important unless mothers feel sick, women's negative experiences with such care, and the belief that postnatal care is available only for babies (47).

Our study shows that occupation, wealth quintile, and ethnicity associate with adequate use of postnatal care services. Women working in the service sector were nearly five times more likely to receive adequate postnatal care than women engaged in agriculture. Women in the fourth and fifth (richest) wealth quintiles were seven times more likely to receive adequate postnatal care compared to those in the poorest quintile, concurring with studies from India and Bangladesh (17, 48).

### Strengths and limitations

Since its establishment at the end of 2010, the JD-HDSS has provided an important sampling frame for various health research studies. Our follow-up study includes demographic and health parameters measured at baseline, thus facilitating comprehensive comparison. Additionally, we report data on women's utilization of SBA services in this peri-urban setting of Nepal, whereas previous published studies focused mostly on rural areas.

Although established HDSS usually conduct annual update rounds (49), we conducted the second round update 2 years after the first survey. This factor might have limited the monitoring of demographic and health events in the JD-HDSS.

Our morbidity data might exhibit bias because we based our measurement on participants’ recall of such events, affecting not only baseline but also follow-up measurements. Comparison with baseline was not possible for some health indicators (e.g. alcohol consumption, SBA use) because they were introduced during the follow-up survey. Nevertheless, comparisons of these indicators will be possible in future follow-up surveys.

The relatively small number of women in the JD-HDSS who had delivered a baby during the prior two years may have influenced the strength of association between use of SBA services and background variables.

## Conclusions

In the JD-HDSS, high in-migration has increased the total population and the number of households. Most health indicators in this peri-urban community exceed the national average. The major morbidity conditions continue to be respiratory diseases, fever, gastrointestinal problems, and bone and joint problems. Our follow-up survey showed an increasing proportion of accidents and injuries and a decreasing proportion of non-communicable diseases. Further investigation of reasons for the increased proportion of accidents and injuries is recommended for their timely prevention.

Most women (90%) accessed institutional delivery and received adequate antenatal care services, but only 13.2% received adequate postnatal care. Availability of transport and use of antenatal care services were associated positively with the use of institutional delivery services.

## References

[1] Byass P (2010). The imperfect world of global health estimates. PLoS Med.

[2] Byass P, Sankoh O, Tollman SM, Hogberg U, Wall S (2011). Lessons from history for designing and validating epidemiological surveillance in uncounted populations. PLoS One.

[3] Aryal U, Vaidya A, Shakya-Vaidya S, Petzold M, Krettek A (2012). Establishing a health demographic surveillance site in Bhaktapur district, Nepal: initial experiences and findings. BMC Res Notes.

[4] Shrestha SS, Shrestha S, Biddlecom AE (2002). The Household Registration System: methods and issues in collecting continuous data on demographic events: Research Report No. 02-517.

[5] Ye Y, Wamukoya M, Ezeh A, Emina JB, Sankoh O (2012). Health and demographic surveillance systems: a step towards full civil registration and vital statistics system in sub-Sahara Africa?. BMC Public Health.

[6] Aryal UR, Petzold M, Bondjers G, Krettek A (2014). Correlates of smoking susceptibility among adolescents in a peri-urban area of Nepal: a population-based cross-sectional study in the Jhaukhel-Duwakot health demographic surveillance site. Glob Health Action.

[7] Vaidya A, Aryal UR, Krettek A (2013). Cardiovascular health knowledge, attitude and practice/behavior in an urbanizing community of Nepal: a population-based cross-sectional study from Jhaukhel-Duwakot health demographic surveillance site. BMJ Open.

[8] Oli N, Vaidya A, Subedi M, Krettek A (2014). Experiences and perceptions about causes and prevention of cardiovascular disease among people with cardiometabolic conditions: findings of in-depth interviews from a semi-urban Nepalese community. Glob Health Action.

[9] Family Health Division (2006). National policy on skilled birth attendants.

[10] New Era (1996). Nepal Family Health Survey.

[11] New Era and Macro International Inc (2007). Trends in demographic and reproductive health indicators in Nepal 2006, further analysis of the 1996, 2001, and 2006. Demographic and Health Survey Data.

[12] Ministry of Health and Population (MoHP) [Nepal], New Era, ICF International Inc (2012). Nepal Demographic and Health Survey 2011.

[13] Sie A, Louis VR, Gbangou A, Muller O, Niamba L, Stieglbauer (2010). The health and demographic surveillance system (HDSS) in Nouna, Burkina Faso, 1993–2007. Glob Health Action.

[14] Ghosh S, Barik A, Majumder S, Gorain A, Mukherjee S, Mazumdar S (2015). Health and demographic surveillance system profile: the Birbhum population project (Birbhum HDSS). Int J Epidemiol.

[15] Choulagai B, Onta S, Subedi N, Mehata S, Bhandari GP, Poudyal A (2013). Barriers to using skilled birth attendants’ services in mid- and far-western Nepal: a cross-sectional study. BMC Int Health Hum Rights.

[16] Onta S, Choulagai B, Shrestha B, Subedi N, Bhandari GP, Kretek A (2014). Perceptions of users and providers on barriers to utilizing skilled birth care in mid- and far-western Nepal: a qualitative study. Glob Health Action.

[17] Singh PK, Rai RK, Alagarajan M, Singh L (2012). Determinants of maternity care services utilization among married adolescents in rural India. PLoS One.

[18] Dhakal S, van Teijlingen E, Raja EA, Dhakal KB (2011). Skilled care at birth among rural women in Nepal: practice and challenges. J Health Popul Nutr.

[19] Mayhew M, Hansen PM, Peters DH, Edward A, Singh LP, Dwivedi V (2008). Determinants of skilled birth attendant utilization in Afghanistan: a cross-sectional study. Am J Public Health.

[20] Rutstein SO, Johnson K The DHS wealth index, DHS comparative reports no 6 (2004).

[21] Bursac Z, Gauss CH, Williams DK, Hosmer DW (2008). Purposeful selection of variables in logistic regression. Source Code Biol Med.

[22] Central Bureau of Statistics (2014). National population and housing census 2011 (Village Development Committee/Municipality), Vol. 6.

[23] World Health Organization (2006). Pregnancy, childbirth, postpartum and newborn care: a guide for essential practice.

[24] Department of Health Services (2012). Annual Report (2010/2011).

[25] Central Bureau of Statistics (2014). Population monograph of Nepal, Vol. I.

[26] Central Bureau of Statistics (2014). Population monograph of Nepal, Vol. II.

[27] Evance I, Godfrey M, Honorati M, Kathleen K (2013). Causes and risk factors for maternal mortality in rural Tanzania – case of Rufiji health and demographic surveillance site (HDSS). Afr J Reprod Health.

[28] Nepal Health Research Council (2009). Epidemiological study on injury and violence in Nepal.

[29] Bhandari GP, Angdembe MR, Dhimal M, Neupane S, Bhusal C (2014). State of non-communicable diseases in Nepal. BMC Public Health.

[30] Vaidya A, Pokharel PK, Nagesh S, Karki P, Kumar S, Majhi S (2009). Prevalence of coronary heart disease in the urban adult males of eastern Nepal: a population-based analytical cross sectional study. Indian Heart J.

[31] Maskey A, Sayami A, Pandey MR (2003). Coronary artery disease: an emerging epidemic in Nepal. J Nepal Med Assoc.

[32] Singh DL, Bhattarai MD (2003). High prevalence of diabetes and impaired fasting glycemia in urban Nepal. Diabet Med.

[33] Binu VS, Subba SH, Menezes RG, Kumar G, Ninan J, Rana MS (2010). Smoking among Nepali youth – prevalence and predictors. Asian Pac J Cancer Prev.

[34] Poudel S, Gurung DK (2013). Prevalence of smoking and perceived health problems among male population of Dharan municipality. J Kathmandu Med Coll.

[35] World Health Organization (2004). WHO Global Status Report on Alcohol 2004.

[36] Niraula SR, Jha N, Shyangwa PM (2013). Alcohol consumption among women in a district of Eastern Nepal. Health Renaissance.

[37] Simkhada B, van Teijlingen E, Porter M, Simkhada P (2008). Factors affecting the utilization of antenatal care in developing countries: systematic review of the literature. J Adv Nurs.

[38] Abosse Z, Woldie M, Ololo S (2010). Factors influencing antenatal care service utilization in Hadiya Zone. Ethiop J Health Sci.

[39] Manithip C, Sihavong A, Edin K, Wahlstrom R, Wessel H (2011). Factors associated with antenatal care utilization among rural women in Lao People's Democratic Republic. Matern Child Health J.

[40] Edmonds JK, Paul M, Sybley L (2012). Determinants of place of birth decisions in uncomplicated childbirth in Bangladesh: an empirical study. Midwifery.

[41] Gabrysch S, Campbell OMR (2009). Still too far to walk: literature review of the determinants of delivery service use. BMC Pregnancy Childbirth.

[42] Shrestha SK, Banu B, Khanom K, Ali L, Thapa N, Stray-Pedersen B (2012). Changing trends on the place of delivery: why do Nepali women give birth at home?. BMC Reprod Health.

[43] Turkmani S, Currie S, Mungia J, Assefi N, Rahmanzai AJ, Azfar P (2013). ‘Midwives are the backbone of our health system’: lessons from Afghanistan to guide expansion of midwifery in challenging settings. Midwifery.

[44] Agha S, Carton TW (2011). Determinants of institutional delivery in rural Jhang, Pakistan. Int J Equity Health.

[45] Population Reference Bureau (2007). Postnatal care: a critical opportunity to save mothers and newborns.

[46] Paudel M, Khanal V, Acharya B, Adhikari M (2013). Determinants of postnatal service utilization in a western district of Nepal: community based cross sectional study. J Women's Health Care.

[47] Tesfahun F, Worku W, Mazengiya F, Kifle M (2014). Knowledge, perception and utilization of postnatal care of mothers in Gondar Zuria District, Ethiopia: a cross-sectional study. Matern Child Health J.

[48] Rahman MM, Hague SE, Zahan MS (2011). Factors affecting the utilisation of postpartum care among young mothers in Bangladesh. Health Soc Care Community.

[49] Kahn K, Collinson MA, Gomez-Olive FX, Obed M, Twine R, Mee P (2012). Profile: Agincourt health and socio-demographic surveillance system. Int J Epidemiol.

